# Simultaneous entanglement swapping of multiple orbital angular momentum states of light

**DOI:** 10.1038/s41467-017-00706-1

**Published:** 2017-09-21

**Authors:** Yingwen Zhang, Megan Agnew, Thomas Roger, Filippus S. Roux, Thomas Konrad, Daniele Faccio, Jonathan Leach, Andrew Forbes

**Affiliations:** 1 CSIR National Laser Centre, PO Box 395, Pretoria, 0001 South Africa; 2 IPaQS, SUPA, Heriot-Watt, Edinburgh, EH14 4AS UK; 3 School of Physics, University of Witwatersrand, Johannesburg, 2000 South Africa; 4 School of Chemistry and Physics, University of KwaZulu-Natal, Private Bag X54001, Durban, 4000 South Africa; 5 National Institute of Theoretical Physics, University of KwaZulu-Natal, Private Bag X54001, Durban, 4000 South Africa; 6 Present Address: Department of Physics, University of Ottawa, 150 Louis Pasteur, Ottawa, Ontario, K1N 6N5 Canada; 7 National Metrology Institute of South Africa, Meiring Naude Road, Pretoria, South Africa

## Abstract

High-bit-rate long-distance quantum communication is a proposed technology for future communication networks and relies on high-dimensional quantum entanglement as a core resource. While it is known that spatial modes of light provide an avenue for high-dimensional entanglement, the ability to transport such quantum states robustly over long distances remains challenging. To overcome this, entanglement swapping may be used to generate remote quantum correlations between particles that have not interacted; this is the core ingredient of a quantum repeater, akin to repeaters in optical fibre networks. Here we demonstrate entanglement swapping of multiple orbital angular momentum states of light. Our approach does not distinguish between different anti-symmetric states, and thus entanglement swapping occurs for several thousand pairs of spatial light modes simultaneously. This work represents the first step towards a quantum network for high-dimensional entangled states and provides a test bed for fundamental tests of quantum science.

## Abstract (web summary)

Entanglement swapping in high dimensions requires large numbers of entangled photons and consequently suffers from low photon flux. Here the authors demonstrate entanglement swapping of multiple spatial modes of light simultaneously, without the need for increasing the photon numbers with dimension.

## Introduction

An integral part of a quantum repeater is the ability to entangle two systems that have not interacted, a process referred to as entanglement swapping^1–7^. In optics, it is accomplished by interfering two photons via Hong–Ou–Mandel (HOM) interference^8–11^, each from a different entangled pair, in such a way that their remote partners become mutually entangled. This allows the establishment of entanglement between two distant points without requiring single photons to travel the entire distance, thus reducing the effects of decay and loss.

While quantum communication has largely been demonstrated using two-level systems—qubits—to carry information, the use of high-dimensional systems allows more information to be encoded per particle. One way to accomplish this is to encode the information in the orbital angular momentum (OAM) of a photon. It is routinely possible to obtain OAM states entangled in very high dimensions^12–16^, and entanglement of OAM is easily produced via spontaneous parametric downconversion (SPDC)^17, 18^, making OAM an ideal method to increase information capacity^19, 20^. Other high-dimensional systems that could increase information capacity include time bins^21^, the path degree of freedom in waveguides^22^ and hybrid entanglement^23–26^. Recently, a number of multi-photon OAM experiments have been reported, including a demonstration of four-photon entanglement^27^ and the creation of Greenberger–Horne–Zeilinger states^28^. However, realising entanglement swapping and teleportation in high dimensions has been thought to require increasing the photon number with dimension^29, 30^, a prohibitive constraint due to the low count rates associated with many-photon entanglement experiments.

In this work, we perform the implementation of entanglement swapping of spatial states of light. We use photons entangled in the OAM degree of freedom and transfer entanglement from one pair of entangled photons to another, even though the final entangled pair have not interacted with each other. We present results for swapped entanglement in six two-dimensional subspaces. Four of these subspaces did not show entanglement prior to the entanglement swapping. We combine these six subspaces into a four-dimensional mixed state that is representative of the final state in high dimensions. We outline entanglement purification schemes to convert this mixed state into a pure high-dimensional state, allowing scalability of our approach to any dimension without the need for additional ancillary photons, thus providing an approach towards high-dimensional, long-distance secure quantum communication.

## Results

### Theory

Our goal is to establish entanglement between two parties that have not interacted. We start with two pairs of entangled photons. The first pair is an entangled state shared by Alice (A) and Bob (B); the second pair is an entangled state shared by Charlie (C) and Daisy (D). Successful swapping corresponds to transferring the entanglement from A and B to A and D, and is equivalent to the teleportation of the state of photon B to photon D.

We generate entangled photons using SPDC in 1-mm-thick β-barium borate (BBO) crystals. We use two crystals to generate two pairs, one in each crystal. Both crystals are pumped with ≈350 mW of light at a wavelength of 404 nm, resulting in two pairs of photons centred at 808 nm. The state at the output of each crystal is entangled in multiple degrees of freedom, including horizontal and vertical position, radial and orbital angular momentum, etc., resulting in a large multi-dimensional state with several thousand entangled modes^16, 31^. When we consider only the OAM index, the state is given by^17^

1 $${\left| {{\Psi }} \right\rangle _{ij}} = \mathop {\sum}\limits_{\ell = 1}^\infty {c_\ell }{\left| {{{\Psi }}_{ - \ell \ell }^ + } \right\rangle _{ij}} + {c_0}{\left| 0 \right\rangle _i}{\left| 0 \right\rangle _j},$$

where the squared modulus of the complex coefficients, ${\left| {{c_\ell }} \right|^2}$, is the probability to find both photons *i* and *j* in the entangled state $\left| {{{\Psi }}_{ - \ell \ell }^ + } \right\rangle$. The entangled state is given by ${\left| {{{\Psi }}_{\ell k}^ \pm } \right\rangle _{ij}} = \left( {{{\left| \ell \right\rangle }_i}{{\left| k \right\rangle }_j} \pm {{\left| k \right\rangle }_i}{{\left| \ell \right\rangle }_j}} \right){\rm{/}}\sqrt 2$, where $\left| \ell \right\rangle$ represents a photon with OAM $\ell \hbar$.

In the experimental aspect of this work, we focus only on the $\ell = \pm 1$ and $\ell = \pm 2$ subspaces, though the analysis can be easily extended to include the entire multi-dimensional state generated by the crystal. Considering the output of both crystals together, where the first (second) crystal produces photons A and B (C and D), we have the initial state given by

2 $$\begin{array}{*{20}{l}}\\ {\left| {{\Psi }} \right\rangle } = &\!\!\! {\left( {{c_1}{{\left| {{{\Psi }}_{ - 11}^ + } \right\rangle }_{{\rm{AB}}}} + {c_2}{{\left| {{{\Psi }}_{ - 22}^ + } \right\rangle }_{{\rm{AB}}}}} \right)} \hfill \\ \\ &\!\!\!{} { \otimes \left( {{c_1}{{\left| {{{\Psi }}_{ - 11}^ + } \right\rangle }_{{\rm{CD}}}} + {c_2}{{\left| {{{\Psi }}_{ - 22}^ + } \right\rangle }_{{\rm{CD}}}}} \right).} \hfill \\ \end{array}$$

Note that there is no entanglement between parties A and D. Photons B and C are then incident on a beamsplitter (BS), where they undergo HOM interference. Our recent work^11^ showed that this can act as a specific filter for the spatial modes of light, whereby any antisymmetric input state results in antibunching and a guaranteed coincidence detection. Conditioned on a coincidence between B and C, the two-photon state between photons A and D becomes

3 $$\begin{array}{*{20}{l}}\\ {{\rho _{_{{\rm{AD}}}}}} = \hfill &\!\!\! {{{\cal K}^2}{{\left| {{c_1}} \right|}^4}\left| {{{\Psi }}_{ - 11}^ - } \right\rangle \left\langle {{{\Psi }}_{ - 11}^ - } \right| + {{\cal K}^2}{{\left| {{c_2}} \right|}^4}\left| {{{\Psi }}_{ - 22}^ - } \right\rangle \left\langle {{{\Psi }}_{ - 22}^ - } \right|} \hfill \\ \\ {} \hfill &\!\!\! { + {{\cal K}^2}{{\left| {{c_1}} \right|}^2}{{\left| {{c_2}} \right|}^2}\left( {\left| {{{\Psi }}_{ - 21}^ - } \right\rangle \left\langle {{{\Psi }}_{ - 21}^ - } \right| + \left| {{{\Psi }}_{ - 12}^ - } \right\rangle \left\langle {{{\Psi }}_{ - 12}^ - } \right|} \right.} \hfill \\ \\ {} {} \hfill &\!\!\! { + \left. {\left| {{{\Psi }}_{12}^ - } \right\rangle \left\langle {{{\Psi }}_{12}^ - } \right| + \left| {{{\Psi }}_{ - 1 - 2}^ - } \right\rangle \left\langle {{{\Psi }}_{ - 1 - 2}^ - } \right|} \right),} \hfill \\ \end{array}$$

where ${\cal K}$ is a normalisation factor. The result is a statistical mixture of the antisymmetric states corresponding to all combinations of two OAM values (Supplementary Note 1). Importantly, the state of A and D now contains entanglement. When we consider a particular OAM value, for example $\ell = \pm 1$, we note that the following transformation occurs:

4 $${\left| {{{\Psi }}_{ - \ell \ell }^ + } \right\rangle _{{\rm{AB}}}} \otimes {\left| {{{\Psi }}_{ - \ell \ell }^ + } \right\rangle _{{\rm{CD}}}} \to {\left| {{{\Psi }}_{ - \ell \ell }^ - } \right\rangle _{{\rm{AD}}}} \otimes {\left| {{{\Psi }}_{ - \ell \ell }^ - } \right\rangle _{{\rm{BC}}}}{\kern 1pt} ,$$

indicating a successful swap of entanglement from AB to AD. Note that in addition, the transformation swaps entanglement from CD to BC. However, this entanglement is lost due to the absorption of the photons BC in the detection process.

We also note that entangled states are created that did not exist prior to the BS, i.e. $\left| {{{\Psi }}_{ - 21}^ - } \right\rangle$, $\left| {{{\Psi }}_{ - 12}^ - } \right\rangle$, $\left| {{{\Psi }}_{12}^ - } \right\rangle$ and $\left| {{{\Psi }}_{ - 2 - 1}^ - } \right\rangle$. One can see this as the result of a transcription process where the basis for one of the subsystems in the initial state is replaced by a different basis in the final state. Transcription is commonly performed to produce OAM entanglement from polarisation entanglement by imprinting one OAM ${\ell _1}$ on horizontally polarised light and another OAM ${\ell _2}$ on vertically polarised light^32^. These transcription processes come about because of the different combinations of the terms allowed in four dimensions: the photon pair in BC (as well as the pair in AD, due to conservation of angular momentum) can be projected into one of six antisymmetric states by a coincidence detection after the BS^11, 30^. See Supplementary Fig. 1 for further details and an example of the process.

### Experiment

**Fig. 1:**
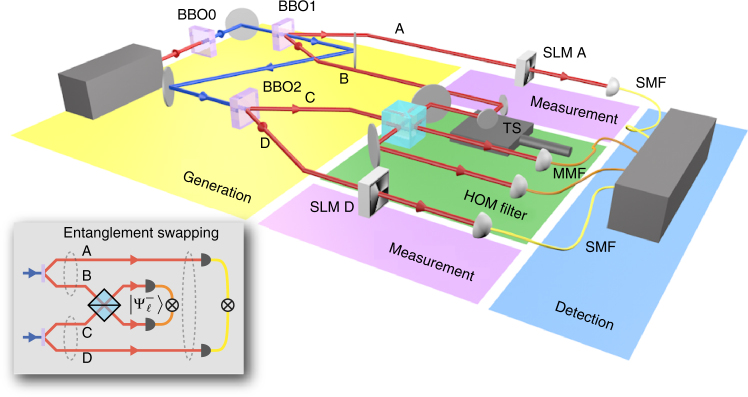
A simplified version of the experimental setup. Generation: the first β-barium borate crystal (BBO0) is pumped by a Ti:Sapphire laser to produce UV light via upconversion. BBO1 produces a downconverted pair A and B; BBO2 produces a downconverted pair C and D. Each is entangled in the state $\left| {{{{\Psi }}^ + }} \right\rangle$. Hong–Ou–Mandel (HOM) filter: the path length of B is adjusted using a translation stage (TS) such that B and C interfere on a beamsplitter; they are projected onto the antisymmetric state when detected in coincidence in the multi-mode fibres (MMFs). Measurement: at this point, photons at A and D become entangled, which we measure using spatial light modulators (SLMs) in combination with single-mode fibres (SMFs). Detection: each photon is detected using a single-photon avalanche detector, and coincidences are determined using a four-way coincidence detection system. *Inset*: a conceptual diagram of entanglement swapping. Entanglement between A and B is transferred to A and D via interference at a beamsplitter and detection in coincidence

In order to verify experimentally that our scheme successfully swaps entanglement from the photons in AB to the photons in AD, the state of photons A and D is determined using projective measurements with a combination of spatial light modulators (SLMs) and single-mode fibres (SMFs). We perform tomography on the state of photons A and D to determine the degree of entanglement (Fig. 1). See ‘Methods’ for further experimental details.

**Fig. 2:**
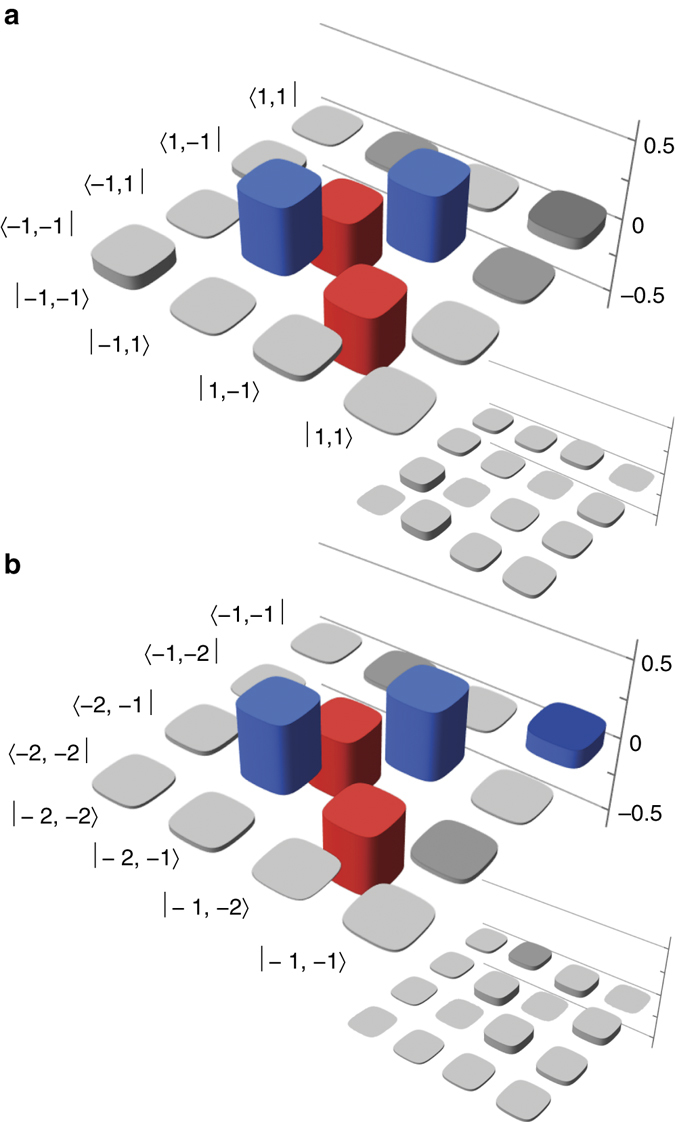
Two-dimensional subspaces. Reconstructed density matrices of the joint state of A and D for **a** $\ell = \pm 1$ and **b** $\ell = - 2, - 1$. Positive values are shown in *blue*, while negative values are shown in *red*; *grey bars* indicate the absolute value is <0.1. The main images show the real part of the state, while the *insets* show the imaginary part

We perform full two-qubit tomography on the $\ell = \pm 1$; $\ell = \pm 2$; $\ell = 2, - 1$; $\ell = 2,1$; $\ell = 1, - 2$ and $\ell = 1,2$ subspaces. We display the reconstructed density matrices of $\ell = \pm 1$ and $\ell = - 2, - 1$ in Fig. 2, while the other four can be seen in Supplementary Fig. 2.

The fidelity of each state with the ideal state $\left| {{{{\Psi }}^ - }} \right\rangle \left\langle {{{{\Psi }}^ - }} \right|$ is an indicator of the success of the entanglement swapping. The fidelities of our two-dimensional reconstructed states are shown in Table 1; they have an average fidelity of 0.80 ± 0.10. The maximum fidelity in our entanglement swapped states is dictated by the visibility of our HOM dip (see ‘Methods’), which is comparable to the visibility obtained in other experiments^28^.

**Table 1:** Measures of entanglement

**Subspace**	**Fidelity**	**Concurrence**
$\ell = \pm 1$	0.80 ± 0.02	0.67 ± 0.04
$\ell = \pm 2$	0.86 ± 0.04	0.75 ± 0.08
$\ell = - 2, - 1$	0.83 ± 0.04	0.76 ± 0.07
$\ell = - 2,1$	0.77 ± 0.02	0.65 ± 0.05
$\ell = 2, - 1$	0.79 ± 0.07	0.65 ± 0.11
$\ell = 2,1$	0.74 ± 0.04	0.61 ± 0.07
Average	0.80 ± 0.10	0.68 ± 0.18

Fidelity and concurrence for each of the six two-dimensional subspaces

Concurrence is a convenient measure of entanglement for two-dimensional subspaces (see ‘Methods’); nonzero concurrence indicates the existence of entanglement, with unit concurrence indicating maximal entanglement. We find a nonzero concurrence for all of the subspaces we reconstruct, as shown in Table 1; the average is 0.68 ± 0.18. This nonzero concurrence indicates successful swapping in multiple two-dimensional subspaces.

The density matrices above were calculated using background-subtracted count rates (see ‘Methods’). We also provide a full analysis of the density matrices obtained with and without background subtraction for the $\ell = \pm 1$ subspace in Supplementary Note 3. As can be expected, we find a higher fidelity and concurrence (0.80 ± 0.02 and 0.67 ± 0.04) for the density matrix generated using the background-subtracted data as compared to that using the raw data (0.57 ± 0.02 and 0.16 ± 0.05). The data for the remaining subspaces can be found in Supplementary Table 2. One could negate the influence of the background by performing projective measurements in arms B and C with SLMs, but this would result in reduced dimensionality in the final state (Supplementary Note 3).

**Fig. 3:**
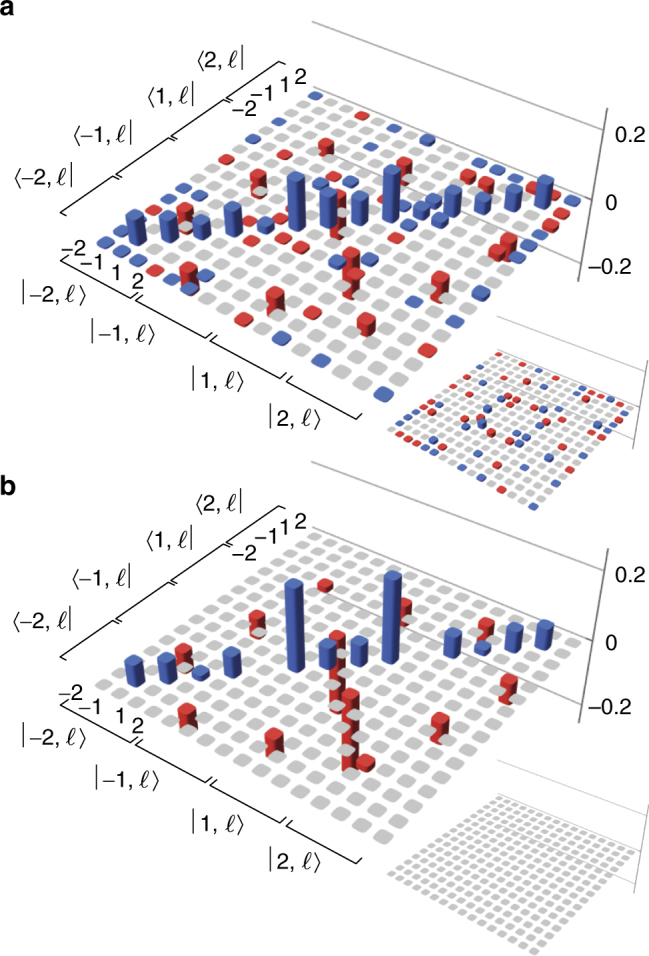
High-dimensional state. Estimated density matrix of the joint state of A and D for the four-dimensional space with $\ell = \pm 1, \pm 2$. **a** The state estimated using the reconstructed density matrices of all six two-dimensional subspaces in Eq. (3). **b** The theoretical prediction using the experimentally observed spiral bandwidth. Positive values are shown in *blue*, while negative values are shown in *red*; *grey* indicates the element is unmeasured in **a** or zero in **b**

In order to estimate the four-dimensional state, we sum the density matrices of the six subspaces together according to Eq. (3). The resulting state is shown in Fig. 3. The elements of the matrix that remain unmeasured are expected to be zero; these do not affect the fidelity of the final state as measurement of the fidelity requires only the diagonal elements and off-diagonal non-zero elements^28^. The fidelity of our estimated state with respect to the state in Eq. (3) is 0.85 ± 0.01, indicating a good overlap between the states.

## Discussion

As our work is the demonstration of entanglement swapping for spatial states, it represents an important step towards realising a quantum repeater for spatial modes of light. Moreover, we note that entanglement swapping implies that teleportation has also taken place. In fact, specific single-photon teleported states have been measured in this work, and these states can be extracted from the two-photon reconstructed states shown in Fig. 2.

In our implementation, the final state between photons A and D is a mixture of all possible two-qubit antisymmetric entangled states $\left| {{{{\Psi }}^ - }} \right\rangle$. Considering the multi-dimensional nature of the light generated by SPDC, we estimate there to be several thousand entangled modes in this state^15, 16^. To experimentally measure this number of modes, one needs to take into account both the OAM and radial indices.

For the future, in contrast to previous work^30^, it is possible to achieve a final state between photons A and D that is pure, i.e., a high-dimensionally entangled state analogous to that of Eq. (1). An additional BBO crystal can be used to up-convert photons B and C^33^. If the up-converted photon is detected in the $\ell = 0$ mode, photons A and D are projected into a pure state. Such a mechanism generates pure high-dimensional entanglement without the need for additional photons. Further details of this mechanism are detailed in Supplementary Note 2.

Furthermore, the present method generates entanglement between modes that were not entangled in the parent photon pairs and thus provides the ability to transcribe entanglement as explained in Supplementary Fig. 2. We believe that the correlations between photons that have not interacted with each other will find applications in remote state engineering, remote ghost imaging and multi-party quantum key distribution.

In conclusion, we have demonstrated the entanglement swapping of OAM states of light. We have confirmed the completion of the entanglement swapping by performing complete tomography of the final entangled pair in multiple two-dimensional OAM subspaces. For all of the subspaces that we consider, we measure a final concurrence greater than zero, indicating that our swap was successful. This result confirms that we have achieved entanglement swapping for multiple OAM subspaces. For each subspace, we obtained an average fidelity of 80% between the reconstructed state and the maximally entangled antisymmetric state. This can be viewed as the first step to building a quantum repeater with spatial modes of light, an essential ingredient for broadband long-distance quantum communication.

## Methods

### Experimental details

**Fig. 4:**
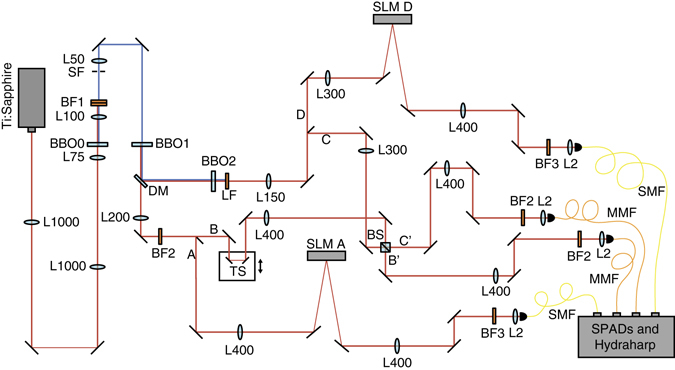
A detailed schematic of the experimental setup. L1000: lens of focal length 1000 mm. L75: lens of focal length 75 mm. BBO0: 0.5-mm-thick β-barium borate crystal. L100: lens of focal length 100 mm. BF1: two consecutive bandpass filters at 405 ± 5 nm. SF: 100-μm circular aperture. L50: lens of focal length 50 mm. BBO1: 1-mm-thick β-barium borate crystal. DM: dichroic mirror. BBO2: 1-mm-thick β-barium borate crystal. LF: longpass filter, cutoff wavelength 750 nm. L150: lens of focal length 150 mm. L300: lens of focal length 300 mm. SLM: spatial light modulator. L400: lens of focal length 400 mm. BF2: bandpass filter at 808 ± 1.5 nm. BF3: bandpass filter at 810 ± 10 nm. L200: lens of focal length 200 mm. TS: translation stage. BS: non-polarising beamsplitter. L2: lens of focal length 2 mm. SMF: single-mode fibre. MMF: multi-mode fibre. SPAD: single-photon avalanche diode

As seen in Fig. 4, our experiment uses a pulsed Ti:sapphire laser (Coherent Chameleon Ultra II) centred at 808 nm, with a pulse width of 140 fs and a repetition rate of 80 MHz. We image the output plane of the laser to the beginning of our setup using two lenses of focal length 1000 mm (L1000). Using a lens of focal length 75 mm (L75), we focus the laser into a 0.5-mm-thick BBO crystal (BBO0). The resultant sum frequency generation produces ≈350 mW of UV light at 404 nm. We focus the upconverted light through a 100-μm circular aperture (spatial filter SF) using a 100-mm lens (L100). The light that passes through the aperture is collimated with a 50-mm lens (L50). The spatial filtering at the aperture ensures that the pump beam used for the downconversion has a Gaussian beam profile. The remaining infrared light is removed using two consecutive bandpass filters BF1 (10-nm width centred at 405 nm).

The UV light is used to pump a 1-mm BBO crystal (BBO1), producing pairs of photons at 808 nm via type-I, near-collinear SPDC. The remaining UV light is deflected using a dichroic mirror (DM), and the downconverted light continues on through a 200-mm lens (L200). It is then split using a D-shaped mirror so that one photon continues on as photon B and the other is reflected as photon A.

Photon B strikes two mirrors on a motorised translation stage (TS) for precise path length adjustment. Photon B then passes through a 400-mm lens (L400) before striking a BS in the image plane of BBO1. Meanwhile, photon A passes through L400 before striking SLM A in the image plane of BBO1. SLM A is imaged to an SMF using L400 and a 2-mm lens (L2).

After being deflected by the DM, the UV light then pumps a second 1-mm BBO crystal (BBO2), after which it is filtered out using longpass filter LF (cutoff wavelength 750 nm). A second pair of photons at 808 nm is produced via SPDC and passes through lens L150. It is then split with a D-shaped mirror so that one photon continues on as photon D and the other is reflected as photon C.

Photon D passes through a 300-mm lens (L300) before striking SLM D in the image plane of BBO2. SLM D is imaged to an SMF using L400 and L2. Photon C passes through L300 before striking the BS in the image plane of BBO2.

**Fig. 5:**
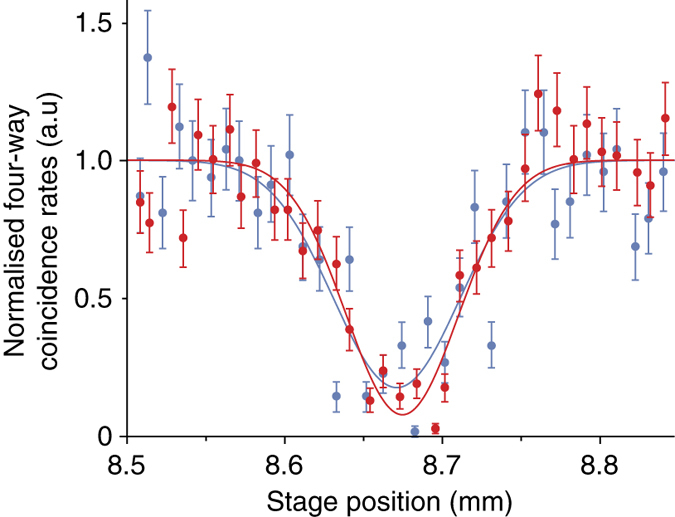
Hong–Ou–Mandel (HOM) interference. HOM dips for photons in the OAM $\ell = \pm 1$ subspace. The *red points* show data when the photons in paths B and C are both in the mode $\left| 1 \right\rangle$; the *blue points* show data when the photons in paths B and C are both in the mode $\frac{1}{{\sqrt 2 }}\left( {\left| 1 \right\rangle + \left| { - 1} \right\rangle } \right)$. We fit a Gaussian curve with a visibility of 0.86 ± 0.04 and 0.70 ± 0.05, respectively. The *error bars* correspond to the standard deviation of the count rate assuming Poisson statistics, and these data are background-subtracted

Here photons B and C undergo HOM interference; the exact position of the HOM interference dip is identified by moving the TS in path B until a minimum in the four-photon coincidence rate is observed. In Fig. 5, we show two HOM dips: the red points indicate when both photons are in the mode $\left| 1 \right\rangle$, while the blue points indicate when both photons are in the mode $\left( {\left| 1 \right\rangle + \left| { - 1} \right\rangle } \right){\rm{/}}\sqrt 2$. After the BS, the new paths B′ and C′ are each imaged to multi-mode fibres (MMFs, core diameter 50 μm) using L400 and L2.

Prior to entering the fibres, each photon encounters a bandpass filter to select a narrow band of wavelengths. BF2, which has a 3-nm spectral width centred at 808 nm, is used in paths B′ and C′ in order to ensure a HOM dip of sufficient width and depth. BF3, which has a 20-nm spectral width centred at 810 nm, is used in paths A and D in order to maximise count rates.

Each of the four fibres is connected to a single-photon avalanche detector (SPAD, Excelitas SPCM-800-14-FC), which is in turn connected to a coincidence detection system (HydraHarp). The average count rate for the $\ell = \pm 1$ subspace is 0.04 counts per second, while the average count rate for the $\ell = \pm 2$ subspace is 0.01 counts per second.

The combined two-dimensional state of photons A and D is determined by displaying holograms of four OAM states on each SLM in turn: $\left| {{\ell _1}} \right\rangle$, $\left| {{\ell _2}} \right\rangle$, $\left( {\left| {{\ell _1}} \right\rangle + \left| {{\ell _2}} \right\rangle } \right){\rm{/}}\sqrt 2$ and $\left( {\left| {{\ell _1}} \right\rangle + i\left| {{\ell _2}} \right\rangle } \right){\rm{/}}\sqrt 2$. Using the 16 resulting measurements, we reconstruct the density matrix using quantum state tomography.

### Spatial light modulators

The OAM of light can be measured with the combination of an SLM and an SMF. An SLM displays a computer-generated hologram of an OAM mode ${\ell _{{\rm{SLM}}}}$; the phase displayed by the SLM is added to that of the incident light. Any reflected light with OAM $\ell = 0$ will successfully couple into the fibre. The detected light then must have had OAM $\ell = - {\ell _{{\rm{SLM}}}}$ prior to striking the SLM.

### Fidelity vs. visibility

The fidelity of a density matrix *ρ* with another density matrix *σ* is

5 $$F = {\rm{Tr}}{\left( {\sqrt {\sqrt \rho \sigma \sqrt \rho } } \right)^{\!\!2}}.$$

Unit fidelity indicates perfect overlap between the states, while zero fidelity indicates no overlap between the states.

The visibility of the HOM dip limits the quality of results. With a visibility of *V*, the entanglement swapping only occurs *V*% of the time. Then (1 − *V*)% of the time, the interference at the BS is unsuccessful, and the resultant four-way coincidences represent uncorrelated noise. Under this assumption, the total two-dimensional state measured is then given by

6 $${\rho _{{\rm{AD}}}} = V\left| {{{{\Psi }}^ - }} \right\rangle \left\langle {{{{\Psi }}^ - }} \right| + (1 - V)\frac{{\Bbb I}}{4},$$

where ${\Bbb I}$ is the identity matrix.

**Fig. 6:**
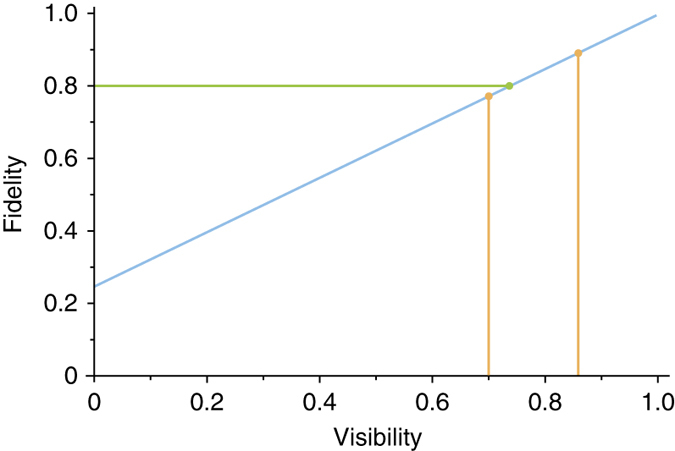
Fidelity vs. visibility. Fidelity of the predicted state with the ideal states as a function of HOM visibility for the $\ell = \pm 1$ subspace. The *green point* corresponds to the measured fidelity (0.80 ± 0.02); the *orange points* correspond to the measured four-way HOM visibility of the data in Fig. 5 (0.86 ± 0.04 and 0.70 ± 0.05). The *blue line* corresponds to the theoretical prediction

The fidelity of the predicted state *ρ*_AD_ with the ideal state $\left| {{{{\Psi }}^ - }} \right\rangle \left\langle {{{{\Psi }}^ - }} \right|$ as a function of visibility is shown in Fig. 6. The measured fidelity of the $\left| {{{\Psi }}_{ - 11}^ - } \right\rangle$ state is shown in green, while the expected fidelities corresponding to our measured visibilities are shown in orange. The measured fidelity is between the two expected fidelities because in reconstructing the full state, we use measurements from both of the measured HOM dip bases.

### Concurrence

The concurrence of a density matrix *ρ* is calculated by first obtaining a matrix

7 $$R = \sqrt {\sqrt \rho \tilde \rho \sqrt \rho } ,$$

where $\tilde \rho = \left( {{\sigma _y} \otimes {\sigma _y}} \right){\rho ^*}\left( {{\sigma _y} \otimes {\sigma _y}} \right)$. Here *σ*_*y*_ represents the Pauli spin matrix and ρ* represents the complex conjugate of *ρ*. The eigenvalues of the matrix *R* are denoted *λ*_1_, *λ*_2_, *λ*_3_ and *λ*_4_ in decreasing order. Then the concurrence of *ρ* is

8 $$C(\rho ) = {\rm{max}}\left( {0,{\lambda _1} - {\lambda _2} - {\lambda _3} - {\lambda _4}} \right).$$

Nonzero concurrence indicates the state is entangled. Unit concurrence indicates a maximally entangled state.

### Background subtraction

*Expected four-way coincidence*: Consider a laser with a repetition rate of *R* pumping a nonlinear crystal to generate an entangled photon pair A and B via SPDC. If $C_{{\rm{AB}}}^\prime$ is the number of coincidence events per second detected between detectors A and B, then $C_{{\rm{AB}}}^\prime$/*R* is the probability that a coincidence event will be detected (or generated, as the probability of detection and generation differ only by a constant factor, the detection efficiency) from a single laser pulse.

Now consider the same laser pulse pumping a second crystal to generate a second pair of photons. The probability to detect/generate these two uncorrelated photon pairs from the same laser pulse, with one pair detected at detectors A and B and the other at C and D, is given by

9 $$\frac{{C_{{\rm{AB}}}^\prime C_{{\rm{CD}}}^\prime }}{{{R^2}}}.$$

Then the rate per second is given by Eq. (9) multiplied by the repetition rate *R*, which gives

10 $$\frac{{C_{{\rm{AB}}}^\prime C_{{\rm{CD}}}^\prime }}{R}.$$

In our experiment a photon pair generated by BBO1 can be detected in coincidence by detectors A&B or A&C, and a second pair generated by BBO2 can be detected by detectors B&D or C&D, so we add all the combinations that can result in coincidence between all four detectors. Therefore the number of four-way coincidence events per second detected from two entangled photon pairs is

11 $$C_{{\rm{4W}}}^\prime = \frac{1}{R}\left( {C_{{\rm{AB}}}^\prime C_{{\rm{CD}}}^\prime + C_{{\rm{AC}}}^\prime C_{{\rm{BD}}}^\prime } \right).$$

*Background of four-way coincidence*: In any experiment measuring coincidences, some detected coincidences will be accidentals, caused simply by two uncorrelated photons arriving at the detectors at the same time. So the detected two-way coincidences $C_{{\rm{AB}}}^\prime$ are the sum of real coincidences *C*_AB_ and accidentals *A*_AB_ = *S*_A_ *S*_B_/*R* where *S*_*i*_ is the number of single counts at detector *i*. So Eq. (11) can be written as

12 $$\begin{array}{*{20}{l}}\\ {C_{4{\rm{W}}}^\prime } = &\!\!\! {\frac{1}{R}\left[ {\left( {{C_{{\rm{AB}}}} + \frac{{{S_{\rm{A}}}{S_{\rm{B}}}}}{R}} \right)\left( {{C_{{\rm{CD}}}} + \frac{{{S_{\rm{C}}}{S_{\rm{D}}}}}{R}} \right)} \right.} \hfill \\ \\ {} {} &\!\!\! {\left. { + \left( {{C_{{\rm{AC}}}} + \frac{{{S_{\rm{A}}}{S_{\rm{C}}}}}{R}} \right)\left( {{C_{{\rm{BD}}}} + \frac{{{S_{\rm{B}}}{S_{\rm{D}}}}}{R}} \right)} \right]} \hfill \\ \\ {} \ \ \ \ = &\!\!\!\!\!\!\ {\frac{1}{R}\left( {{C_{{\rm{AB}}}}{C_{{\rm{CD}}}} + {C_{{\rm{AC}}}}{C_{{\rm{BD}}}}} \right)} \hfill \\ \\ {} \hfill {} \hfill &\!\!\!\! {+ \frac{1}{{{R^2}}}\left( {{C_{{\rm{AB}}}}{S_{\rm{C}}}{S_{\rm{D}}} + {S_{\rm{A}}}{S_{\rm{B}}}{C_{{\rm{CD}}}}} \right.} \hfill \\ \\ {} \hfill &\!\!\!\! {\left. { + {C_{{\rm{AC}}}}{S_{\rm{B}}}{S_{\rm{D}}} + {S_{\rm{A}}}{S_{\rm{C}}}{C_{{\rm{BD}}}}} \right)} \hfill \\ \\ {} \hfill {} \hfill &\!\!\!\! {+ \frac{2}{{{R^3}}}{S_{\rm{A}}}{S_{\rm{B}}}{S_{\rm{C}}}{S_{\rm{D}}}} \hfill \\ \\ {} \ \ \ \ = &\!\!\!\! {{C_{{\rm{4W}}}} + {A_{{\rm{4W}}}}.} \hfill \\ \end{array}$$

We subtract the calculated number of background counts *A*_4W_ from the measured data $C_{4{\rm{W}}}^\prime$ to obtain the actual number of counts *C*_4W_. Occasionally with count rates that are expected to be very low, the measured number of counts is smaller than the expected number of background counts; in this case, we replace the count rate with zero.

### Data availability

The raw data has been deposited in Research Portal (DOI: 10.17861/63d78c64-af80-4792-88d3-782d42ece602)^1^.

## Electronic supplementary material

Supplementary Information
