# Supplementary material for: Simultaneous entanglement swapping of multiple orbital angular momentum states of light
Source: Nat Commun. 2017 Sep 21;8:632. doi: 10.1038/s41467-017-00706-1 (PMC5608840; doi:10.1038/s41467-017-00706-1)
Supplement: Supplementary file 1 — Supplementary Information [file 41467_2017_706_MOESM1_ESM.pdf]

SUPPLEMENTARY NOTES

Supplementary Note 1: Entanglement swapping in high dimensions

The state of the two photon pairs produced by spontaneous parametric downconversion (SPDC) in paths A, B and C, D, respectively, can be written as

$$|\psi_0\rangle = \left(c_0 \left|0\right\rangle_{\mathrm{A}} \left|0\right\rangle_{\mathrm{B}} + \sum_n c_n \left|\Psi^+_{n\overline{n}}\right\rangle_{\mathrm{AB}}\right) \otimes \left(c_0 \left|0\right\rangle_{\mathrm{C}} \left|0\right\rangle_{\mathrm{D}} + \sum_m c_m \left|\Psi^+_{m\overline{m}}\right\rangle_{\mathrm{CD}}\right),$$

where c_n represents complex coefficients, we use the notation $\overline{n} := -n$ and

$$\left|\Psi^{\pm}_{\ell\ell'}\right\rangle := \frac{1}{\sqrt{2}} \left(|\ell\rangle |\ell'\rangle \pm |\ell'\rangle |\ell\rangle\right) \tag{1}$$

denote symmetric and antisymmetric Bell states with a plus and a minus sign, respectively.

The photons in path B and path C are subjected to a $50:50$ beam splitter which superposes the two beams. The inversion of the helicity of the OAM modes, $\ell \to -\ell$, upon reflection in the beam spitter is compensated by two additional reflections employing a mirror in path B before and another one in path C behind the beam splitter. The action of the beam splitter, combined with both mirrors, is thus characterised by the transformation rules

$$|\ell\rangle_{\mathrm{B}} \to \frac{1}{\sqrt{2}} \left(|\ell\rangle_{\mathrm{C}} - |\ell\rangle_{\mathrm{B}}\right) \tag{2}$$

$$|\ell\rangle_{\mathrm{C}} \to \frac{1}{\sqrt{2}} \left(|\ell\rangle_{\mathrm{B}} + |\ell\rangle_{\mathrm{C}}\right), \tag{3}$$

where we denote the original before the beam splitter and the reflected path thereafter by the same letter; accordingly a photon in the input port B exits in path B upon reflection and in path C upon transmission.

Using these transformation rules on the input state in Supplementary Equation (1), and under the condition that each output path of the beam splitter contains a single photon, we obtain a state after the beam splitter that only consists of antisymmetric photon pairs. For d-dimensions, one can express it as

$$\begin{aligned} |\psi_1\rangle =& \mathcal{K} \Bigg[\sum_{n=1}^{N} c_n^2 \left|\Psi^-_{n\overline{n}}\right\rangle_{\mathrm{AD}} \left|\Psi^-_{n\overline{n}}\right\rangle_{\mathrm{BC}} - \sum_{n=1}^{N} c_0 c_n \left(\left|\Psi^-_{0n}\right\rangle_{\mathrm{AD}} \left|\Psi^-_{0\overline{n}}\right\rangle_{\mathrm{BC}} + \left|\Psi^-_{0\overline{n}}\right\rangle_{\mathrm{AD}} \left|\Psi^-_{0n}\right\rangle_{\mathrm{BC}}\right) \\ &- \sum_{m\neq n=1}^{N} c_m c_n \left(\left|\Psi^-_{n\overline{m}}\right\rangle_{\mathrm{AD}} \left|\Psi^-_{\overline{n}m}\right\rangle_{\mathrm{BC}} + \left|\Psi^-_{\overline{n}m}\right\rangle_{\mathrm{AD}} \left|\Psi^-_{n\overline{m}}\right\rangle_{\mathrm{BC}} + \left|\Psi^-_{nm}\right\rangle_{\mathrm{AD}} \left|\Psi^-_{\overline{n}\overline{m}}\right\rangle_{\mathrm{BC}} + \left|\Psi^-_{\overline{n}\overline{m}}\right\rangle_{\mathrm{AD}} \left|\Psi^-_{nm}\right\rangle_{\mathrm{BC}}\right)\Bigg], \end{aligned} \tag{4}$$

where $\mathcal{K}$ is a normalization constant that compensates for the loss of the terms with two photons in the same output path of the beam splitter and $d = 1 + 2N$. In the case where we only consider $\ell = \pm 1, \pm 2$, we have

$$\begin{aligned} |\psi_1\rangle = \mathcal{K} \Bigg[& c_1^2 \left|\Psi^-_{1\overline{1}}\right\rangle_{\mathrm{AD}} \left|\Psi^-_{1\overline{1}}\right\rangle_{\mathrm{BC}} + c_2^2 \left|\Psi^-_{2\overline{2}}\right\rangle_{\mathrm{AD}} \left|\Psi^-_{2\overline{2}}\right\rangle_{\mathrm{BC}} \\ &- c_1 c_2 \left(\left|\Psi^-_{1\overline{2}}\right\rangle_{\mathrm{AD}} \left|\Psi^-_{\overline{1}2}\right\rangle_{\mathrm{BC}} + \left|\Psi^-_{\overline{1}2}\right\rangle_{\mathrm{AD}} \left|\Psi^-_{1\overline{2}}\right\rangle_{\mathrm{BC}} + \left|\Psi^-_{12}\right\rangle_{\mathrm{AD}} \left|\Psi^-_{\overline{1}\overline{2}}\right\rangle_{\mathrm{BC}} + \left|\Psi^-_{\overline{1}\overline{2}}\right\rangle_{\mathrm{AD}} \left|\Psi^-_{12}\right\rangle_{\mathrm{BC}}\right)\Bigg]. \end{aligned} \tag{5}$$

Simultaneous detection of a single photon in each of the two output ports of a symmetric beam splitter causes a projection onto the antisymmetric component of the input state. The dimension of the corresponding antisymmetric state space is given by the number of ways in which the OAM values of the input space can be combined into pairs of ℓ's to form an antisymmetric state $\left|\Psi^-_{\ell\ell'}\right\rangle$. For example, four OAM values would give six antisymmetric basis states, featuring in the BC-components of the state in Supplementary Equation (4). On the other hand, the antisymmetric basis states also feature as components of the photon pair in AD, because the OAM must sum to zero in each term of the state. In general, considering d OAM levels, we can produce a state of the form given in Supplementary Equation (4) with our setup consisting of $d(d-1)/2$ antisymmetric basis states that involve both photon pairs.

Note that the state expressed in Supplementary Equation (4) represents the Schmidt decomposition of an entangled state, i.e., it has the form

$$|\psi_1\rangle = \sum_i c_i \, |\phi_i\rangle_{\mathrm{AD}} \, |\phi_i\rangle_{\mathrm{BC}} \,, \tag{6}$$

where the Schmidt bases are photon pairs in AD and BC, respectively. So, apart from the entanglement among the different pairs, there is also the maximal entanglement within the pairs between the single photons.

The detection of the photons in paths B and C without measurement of their OAM values results in a statistical mixture of the antisymmetric states as in Supplementary Equation (4). By tracing over the OAM degrees of freedom of the photons in paths B and C, we obtain for d dimensions

$$\begin{aligned} \rho_{\mathrm{AD}} =& \mathcal{K}^2 \Bigg[\sum_{n=1}^{N} |c_n|^4 \, |\Psi^-_{n\bar{n}}\rangle \langle \Psi^-_{n\bar{n}}| + \sum_{n=1}^{N} |c_0|^2 |c_n|^2 \left(|\Psi^-_{0n}\rangle \langle \Psi^-_{0n}| + |\Psi^-_{0\bar{n}}\rangle \langle \Psi^-_{0\bar{n}}| \right) \\ &+ \sum_{m\neq n=1}^{N} |c_m|^2 |c_n|^2 \left(|\Psi^-_{n\bar{m}}\rangle \langle \Psi^-_{n\bar{m}}| + |\Psi^-_{\bar{n}m}\rangle \langle \Psi^-_{\bar{n}m}| + |\Psi^-_{nm}\rangle \langle \Psi^-_{nm}| + |\Psi^-_{\bar{n}\bar{m}}\rangle \langle \Psi^-_{\bar{n}\bar{m}}| \right) \Bigg] . \end{aligned} \tag{7}$$

Restricted to $\ell = \pm 1, \pm 2$, the result reduces to

$$\begin{aligned} \rho_{\mathrm{AD}} =& \mathcal{K}^2 |c_1|^4 \left|\Psi^-_{1\bar{1}}\right\rangle \left\langle \Psi^-_{1\bar{1}}\right| + \mathcal{K}^2 |c_2|^4 \left|\Psi^-_{2\bar{2}}\right\rangle \left\langle \Psi^-_{2\bar{2}}\right| \\ &+ \mathcal{K}^2 |c_1|^2 |c_2|^2 \left(\left|\Psi^-_{1\bar{2}}\right\rangle \left\langle \Psi^-_{1\bar{2}}\right| + \left|\Psi^-_{\bar{1}2}\right\rangle \left\langle \Psi^-_{\bar{1}2}\right| + |\Psi^-_{12}\rangle \langle \Psi^-_{12}| + \left|\Psi^-_{\bar{1}\bar{2}}\right\rangle \left\langle \Psi^-_{\bar{1}\bar{2}}\right| \right) . \end{aligned} \tag{8}$$

The projection onto the antisymmetric space of the photons in B and C transfers entanglement between the systems in A and B to the remote systems in A and D, which were not entangled before. This constitutes entanglement swapping.

Supplementary Note 2: Pure final state

We note that, by using a filter in paths A and D that projects onto any two-dimensional subspace with OAM values $\{\ell, \ell'\}$, one obtains an antisymmetric state $|\Psi^-_{\ell,\ell'}\rangle_{\mathrm{AD}}$, which is maximally entangled. Such a filter in front of the detectors in paths B and C could be used to prepare a particular antisymmetric state remotely in paths A and D. A similar procedure could be exploited for various purposes of quantum communication between three or four parties, such as secure bit commitment or QKD protocols.

By means of particular filters for photons in BC, it is also possible to obtain a pure state with a multitude of entangled levels instead of a mixture in AD. For example, projecting on a superposition of singlet states $\left|\Psi^-_{n\bar{n}}\right\rangle$ in BC results, as shown below, in a superposition of such states in AD. According to Supplementary Equation (4), the state of photons in BC after the beamsplitter reads

$$|\Psi_1\rangle = \sum_{n=1}^{\infty} \alpha_n \, |n\rangle_{\mathrm{AD}} \otimes |n\rangle_{\mathrm{BC}} + \ldots \tag{9}$$

with $|n\rangle \equiv \left|\Psi^-_{n\bar{n}}\right\rangle$ and $\alpha_n \equiv \mathcal{K} c_n^2$, where components $\left|\Psi^-_{n,m}\right\rangle$ with different OAM values $n \neq m$ are not mentioned explicitly. A filter in BC projecting onto the state $|x\rangle \equiv (\sum_{n=1}^{N} |n\rangle)/\sqrt{N}$ leads to

$$\begin{aligned} |\Psi_1\rangle &\to (\mathbb{1}_{\mathrm{AD}} \otimes |x\rangle_{\mathrm{BC}} \langle x|) \, |\Psi_1\rangle \\ &= \frac{1}{\sqrt{N}} \sum_{n=1}^{\infty} \sum_{m=1}^{N} \alpha_n \, |n\rangle_{\mathrm{AD}} \otimes |x\rangle_{\mathrm{BC}} \langle m| \, n\rangle_{\mathrm{BC}} \\ &= \left(\frac{1}{\sqrt{N}} \sum_{n=1}^{N} \alpha_n \, |n\rangle_{\mathrm{AD}} \right) \otimes |x\rangle_{\mathrm{BC}} . \end{aligned} \tag{10}$$

The resulting state of the photons in AD, $\sum_{n=1}^{N} \tilde{\alpha}_n \, |n\rangle \equiv \sum \tilde{\alpha}_n \left|\Psi^-_{n\bar{n}}\right\rangle$ (with normalised coefficients $\tilde{\alpha}_n = c_n^2 / \sqrt{\sum_n |c_n^2|^2}$), is a pure entangled state of Schmidt rank N. Such a filter could be realised, e.g., by parametric up-conversion of the photon pair in AD to a photon of double the frequency (the inverse process to SPDC) and subsequent measurement of its OAM, conditioning on the OAM value $\ell = 0$.

Supplementary Note 3: Background subtraction

In our experiment, we use spatial light modulators (SLMs) and single-mode fibres to detect photons in paths A and D and multi-mode fibres to detect photons in paths B and C. The use of multi-mode fibres is so that we detect within the 4-dimensional space spanning the modes $\{\ell = -2, \ell = -1, \ell = +1, \ell = +2\}$ in an unrestricted fashion, i.e. we do not select out a subspace in paths B and C. The use of additional SLMs and single-mode fibres, rather than multi-mode fibres, would enable a choice of which particular space we detect in, but it would also remove the ability to observe multiple subspaces at once to obtain a high-dimensional state.

A consequence of the multi-mode fibres is, however, that the rate of the single-photon detection events at detectors B and C is significantly higher than that recorded at detectors A and D. Supplementary Table I provides representative single-channel and coincidence count rates recorded for the $\ell = \pm 1$ and $\ell = \pm 2$ subspaces. The B and C single-channel rates recorded with the multi-mode fibres are on average $\approx$4 times higher than those recorded at A and D. This trend is observed across all the subspaces that we investigate.

The large single-channel rates resulting from the multi-mode fibres contribute to unwanted 4-way coincidence counts that do not participate in any entanglement swapping. However, these unwanted 4-way coincidences that arise from uncorrelated photon detection events (Eq. (12) in the main paper) can be calculated and subtracted off the measured 4-way counts. We can use either the raw counts or the background-subtracted counts to calculate density matrices from which fidelities and concurrences are extracted. Supplementary Figure 3 and Supplementary Table II provide evidence of the impact of the background subtraction on the quality of our entanglement swapping. As can be seen, a higher fidelity and concurrence (0.80 ± 0.10 and 0.68 ± 0.18) is observed for the density matrix generated using the background-subtracted data as compared to that using the raw counts (0.54 ± 0.08 and 0.09 ± 0.14).

This increase in quality when background subtraction is applied is to be expected. If, as mentioned above, we project into the $\ell = \pm 1$ subspace using SLMs and single-mode fibres in paths B and C, the raw count rates would only differ from the background-subtracted rates by $\approx 1\%$. This in turn would result in a very small difference between the two different density matrices, and high fidelities and concurrences would be observed in both cases.

SUPPLEMENTARY FIGURES

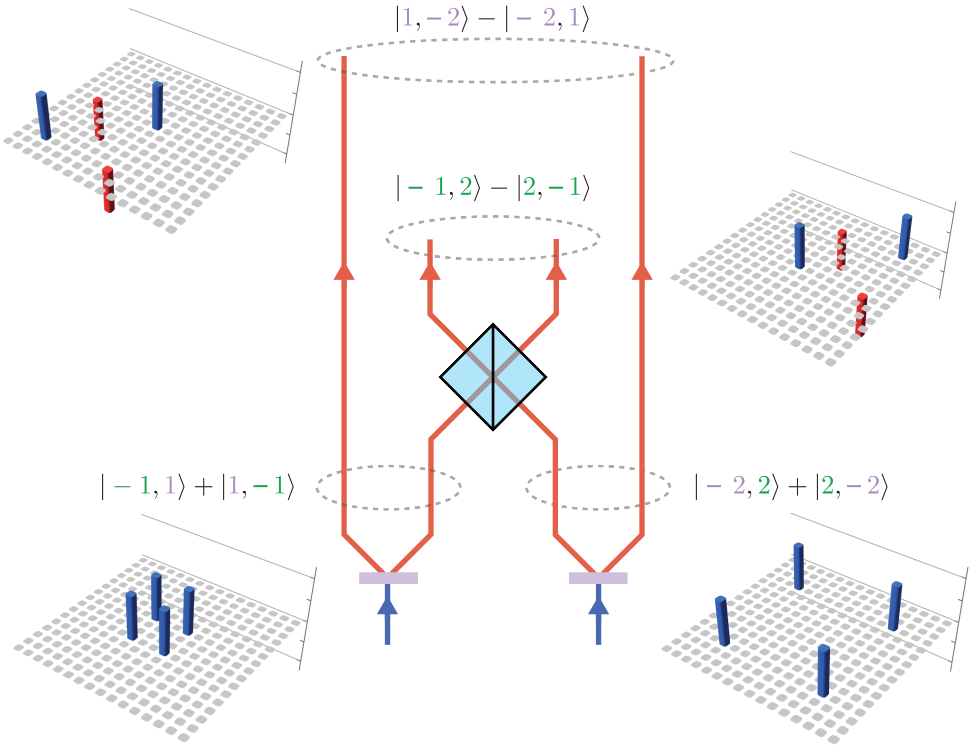

Supplementary Figure 1. **Example of the transcription process.** We start with the entangled states $\left|\Psi^{+}_{-11}\right\rangle_{\mathrm{AB}}$ and $\left|\Psi^{+}_{-22}\right\rangle_{\mathrm{CD}}$. After the beamsplitter and projection onto the appropriate anti-symmetric state $\left|\Psi^{-}_{-12}\right\rangle$, the state between photons A and D is $\left|\Psi^{-}_{-21}\right\rangle_{\mathrm{AD}}$. The OAM values shown in green become the OAM values in the state projected onto B and C. The OAM values shown in purple become the OAM values in the state between photons A and D. States are shown without normalisation for clarity. The density matrices show the corresponding maximally entangled states.

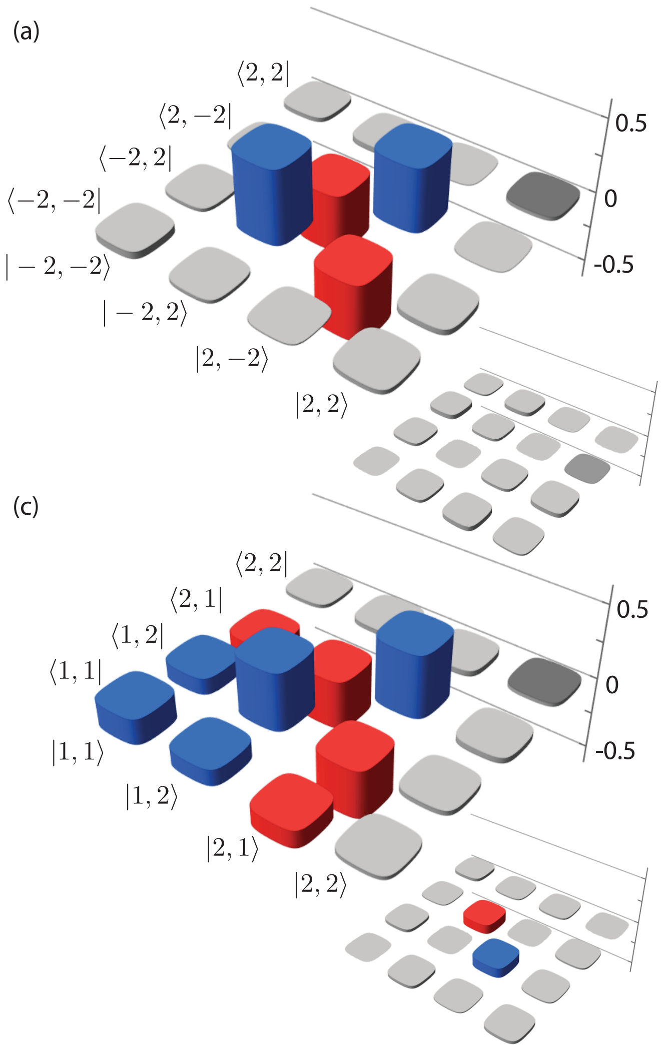

Supplementary Figure 2. **Two-dimensional subspaces.** Reconstructed density matrices of the joint state of A and D for (a) $\ell = \pm 2$, (b) $\ell = -2, 1$, (c) $\ell = 2, 1$, and (d) $\ell = 2, -1$. Positive values are shown in blue, while negative values are shown in red; grey bars indicate the absolute value is less than 0.1. The main images show the real part of the state, while the insets show the imaginary part.

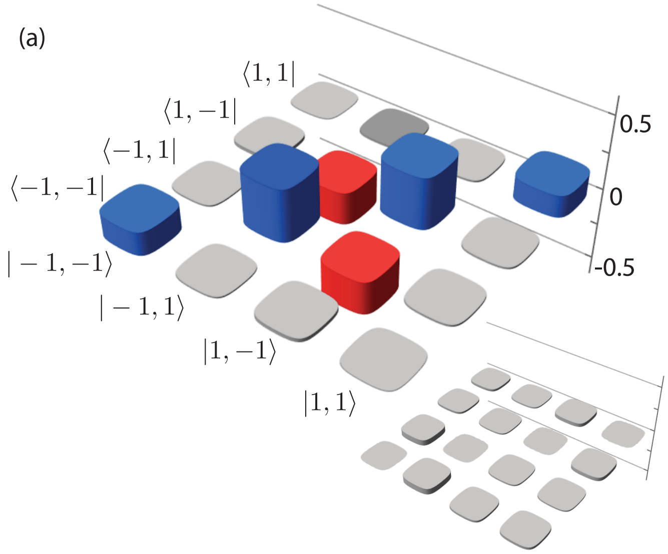

Supplementary Figure 3. **Background subtraction.** Density matrices reconstructed using (a) the raw counts and (b) the background-subtracted counts for the $\ell = \pm 1$ subspace.

SUPPLEMENTARY TABLES

Supplementary Table I. **Raw count rates.** Representative count rates for the $\ell = \pm 1$ and $\ell = \pm 2$ subspaces. The background count rate calculated using Eq. (12) in the main text is also shown. All rates are in counts per second.

Subspace	S_{A}	S_{B}	S_{C}	S_{D}	C_{AB}	C_{AC}	C_{BD}	C_{CD}	Expected raw 4-way counts	Background-subtracted 4-way counts	Accidental 4-way counts
$\ell = \pm 1$	77 000	280 000	280 000	64 000	1800	1500	1700	1500	0.07	0.05	0.02
$\ell = \pm 2$	70 000	280 000	280 000	61 000	1000	900	1000	900	0.02	0.01	0.01

Supplementary Table II. **Fidelities and concurrences.** Fidelity and concurrence for each of the six two-dimensional subspaces.

	BS		Raw	
Subspace	Fidelity	Concurrence	Fidelity	Concurrence
$\ell = \pm 1$	0.80 ± 0.02	0.67 ± 0.04	0.57 ± 0.02	0.16 ± 0.05
$\ell = \pm 2$	0.86 ± 0.04	0.75 ± 0.08	0.50 ± 0.03	0.01 ± 0.05
$\ell = -2, -1$	0.83 ± 0.04	0.76 ± 0.07	0.58 ± 0.03	0.24 ± 0.07
$\ell = -2, 1$	0.77 ± 0.02	0.65 ± 0.05	0.49 ± 0.02	0.03 ± 0.04
$\ell = 2, -1$	0.79 ± 0.07	0.65 ± 0.11	0.50 ± 0.05	0.05 ± 0.08
$\ell = 2, 1$	0.74 ± 0.04	0.61 ± 0.07	0.49 ± 0.03	0.05 ± 0.05
Average	0.80 ± 0.10	0.68 ± 0.18	0.54 ± 0.08	0.09 ± 0.14
